# Impact of Color and Polarity on Visual Resolution with Varying Contrast Ratios and Different Text Backgrounds

**DOI:** 10.18502/jovr.v17i2.10793

**Published:** 2022-04-29

**Authors:** Ai-Hong Chen, Nurulain Muhamad

**Affiliations:** ^1^Optometry, Faculty of Health Sciences, Universiti Teknologi MARA, Cawangan Selangor, Kampus Puncak Alam, Malaysia

**Keywords:** Color; Contrast, Polarity, Text-background, Visual Resolution

## Abstract

**Purpose:**

To assess the impact of color and polarity in predicting the changes of visual resolution for different text backgrounds with increasing contrast ratios.

**Methods:**

Text-background designs of eight contrast ratios (0.15, 0.30, 0.47, 0.52, 0.57, 0.60, 0.70, and 0.78) and two text polarities (positive; black text and negative; white text) were compared with and without the presence of background color (blue, green, orange, and red). The visual resolution was measured in logMAR using Landolt C. The rate of changes in visual resolution measurements was analyzed using linear regression as contrast ratios increased with and without background color.

**Results:**

Visual resolution varied significantly with and without the background color element under both polarity investigations (*P* < 0.05). Contrast ratio accounts for 77.4% of the variation within the visual resolution measurement with a color background [F ${}_{\left(1,6\right)}$ = 20.76, *P* < 0.01]. Contrast ratio accounts for 97.16% of the variation in visual resolution measurements without a color background [F ${}_{\left(1,6\right)}$ = 205.63, *P* < 0.01].

**Conclusion:**

As contrast decreases, color plays a more significant role than the non-color factor in the resolution of fine details in both polarities as it influences the visual resolution outcome which is reflected in the measurements in logMAR units.

##  INTRODUCTION

In any environment, contrast is the element that influences the visibility of an object. Contrast is the manifestation of differences in attributes such as luminance and color of a visual object seen simultaneously.^[1,3]^ The changes in luminance due to the difference between two areas (e.g., between an object and its immediate background) is known as the contrast ratio, which calculates the ratio between the two specified areas.^[4,5]^ Contrast aids vision by providing good legibility of the objects seen.^[6]^ Without a sufficient amount of contrast, the stimulus for vision is affected. Colored objects viewed on a colored background present a color contrast as well as a luminance contrast.^[7,8]^ High contrast is the primary determinant factor for maintaining compelling reading.^[9,10]^ Luminance contrast alone is sufficient to maintain visual performance but color alone is not enough.^[7]^ However, low contrast and mid-range wavelength (yellow) color reduce visual acuity.^[11]^ Text-background color seems to improve contrast perception.^[11,12]^

The effect of text-background color on visual functions, such as reading speed,^[7,8]^ visual search,^[10]^ viewing distance,^[13]^ legibility,^[14,15]^ and polarity^[16,17,18]^ has been studied extensively. However, the influence of color and polarity on readability remains debatable. Some researchers reported better legibility of lighter letters on a darker background than the traditional darker letter on a lighter background in non-color and colored backgrounds.^[16,18]^ Contradictorily, others found the darker target on the lighter background provided better visual performance by enhancing the legibility of the target as compared to a lighter target on a darker background.^[19,20,21]^ The positive and negative polarity of the achromatic stimulus have a negligible effect on legibility in high-contrast ratio conditions.^[11,22]^ Since color impacts visual acuity and is contrast-dependent, this may indicate that the color at multiple contrast ratio levels may affect legibility in varied ways.^[11,12]^ In the present study, we investigated the impact of color and polarity in predicting the changes of visual resolution with different contrast ratio.

##  METHODS

### Experimental Design

**Figure 1:**
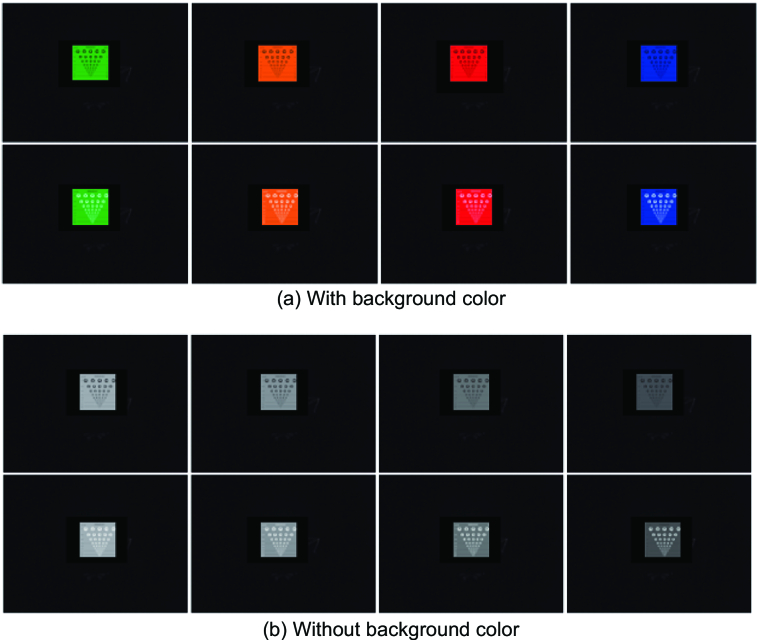
The view of the test scenario at 16 trials, photographed with the subjects' eye level at 4 m working distance: (a) with background color and (b) without background color.

Text-background design of eight contrast ratios (0.15, 0.30, 0.47, 0.52, 0.57, 0.60, 0.70, and 0.78) and two text polarities (positive; black text and negative; white text) were compared with and without the presence of background color (blue, green, orange, and red) [Figure 1]. The dependent variable was visual resolution. The rate of changes in visual resolution measurements with increasing contrast ratios with and without background color was analyzed using linear regression. A repeated measures design was used for this study, whereby the same subjects were tested at all testing conditions. Trials from each condition were randomly interleaved, and the task was always the same.

### Visual Stimuli and Apparatus 

The visual resolution was measured through spatial threshold determination by using four-orientation Landolt C. It was recorded as logMAR (logarithm minimum angle of resolution) using the detection of the gap in a four-orientation Landolt C. A four-orientation design of Landolt C chart was produced on a photo matte surface material and presented to the subjects.^[2,3]^ The chart was internally illuminated with a light-emitting diodes lamp that provided enhanced color properties with reduced flicker.^[24,2,5]^ The matte surface minimized specular reflections to produce an even, diffused, dark-grey background. The experiments were divided into two parts corresponding to the different text designs (black text and white text). The comparison was between backgrounds with and without color in each text-background design. The luminance of the Landolt C was measured using a calibrated Konica Minolta Luminance Meter LS110. The contrast ratio between the text and the background was calculated using the luminance contrast definition of Michelson.^[2,6]^ The formula was constructed using the maximum and minimum luminance of the text and the background [Table 1].

**Figure 2:**
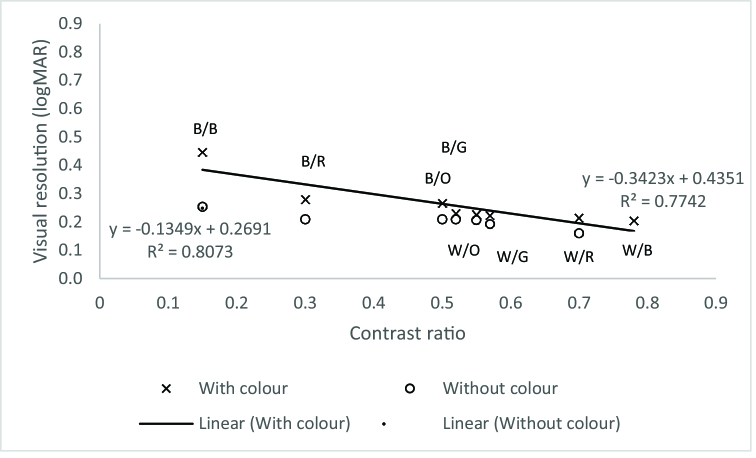
Mean visual resolution measurement (logMAR) as a function of contrast ratio for with and without color of text-background design. B/B, black-on-blue; B/R, black-on-red; B/O, black-on-orange; B/G, black-on-green; W/O, white-on-orange; W/G, white-on-green; W/R, white-on-red; W/B, white-on-blue.

**Table 1:** Summary of luminance information used in the four-position Landolt-C chart designs


**Landolt-C chart designs (Text/Background)**	**Luminance, L (cd/m^2^)**	
		**Text**	**Background**
Black/Blue	6.76	9.13
Black/Red	6.76	12.20
Black/Green	6.76	24.55
Black/Orange	6.76	18.67
White/Blue	71.47	9.13
White/Red	71.47	12.20
White/Green	71.47	24.55
White/Orange	71.47	18.67
	
	

### Room Setup and Experimental Procedure 

Our experiment room was well controlled, and detailed with systematic procedures to minimize technical error and learning bias. Well-controlled settings were essential in reducing inter-variation. Unwanted reflectance might cause color interference and veiling luminance that may lead to glare sensation. The wall, floor, and ceiling of the experiment room were covered with black cloth to reduce light reflection and stray light. The calculated reflectance of the room wall and floor were 0.16 and 0.10, respectively. The room illuminance was controlled at 100 lux. The chart was internally illuminated and placed at 4 m from the subject. The midpoint of the chart panel was positioned at eye level (approximately 1.3 m above the floor). The subjects verified the threshold of the legible letter Cs using four orientation approach of the Landolt C charts. Five-minute dark adaptation was allowed at the beginning of each measurement. Instructions regarding the procedure were explained. The Landolt C charts were presented to the subject in random order. The subject was required to give their response by indicating the orientation of the Landolt C gap. The performance was scored and measured as the minimum angle of resolution.

### Participants

This study adhered to the tenets of the Declaration of Helsinki and was approved by the university's Research Ethics Committee. All power calculations were conducted using the GPower analysis program.^[28]^ Given a total sample size of *n* = 31 and assuming α = 0.05, population effects of size *f* = 0.80 (large effects ${}^{[}$ 28 ${}^{]}$ ) could be detected for the independent variables with a probability of 1 – β = 0.95. Thirty-one subjects were young adults (mean age of 22.46 ± 1.85 years) with no known ocular pathology. All participants were screened with a D-15 color vision test to rule out any known color deficiencies. The inclusion criteria were as follows: (i) logMAR 0.20 or better habitual distance visual acuity; (ii) logCS 1.65 or better contrast sensitivity; and (iii) a pass with a circular result diagram of D15 color vision test. In our contrast-color-polarity investigation, a single individual went through a similar process 16 times (representing each chart). The best acuity was obtained for each chart. Subjects were tested with their habitual visual acuity and natural pupil. Simple randomization was used to reduce the learning effect due to repetitive measurements.^[29]^ The technique maintained complete randomness of the assignment of a stimulus presented. The random numbers were generated using the RANDBETWEEN function in Excel.

### Statistical Analysis

The current investigation utilized a within-subject study design, where repeated measures of the same subjects were performed. The data collected from all 31 subjects were analyzed using the SPSS Statistic Software version 20. The visual resolution measurements were analyzed using Friedman's two-way analysis of variance test and linear regression analysis.

##  RESULTS

In the positive polarity investigation (contrast range, 0.15–0.80), the visual resolution of white text was found to vary significantly with background color wavelength [Friedman's two-way analysis of variance test: χ ^2^ = 35.46, *P* < 0.05]. Moreover, the effect of positive polarity on the visual resolution remained significant without color element [Friedman's two-way analysis of variance test: χ ^2^ = 21.00, *P* < 0.05]. In negative polarity investigation, the visual resolution of white text was found to vary significantly with background color wavelength [Friedman's two-way analysis of variance test: χ ^2^ = 16.64, *P* < 0.05]. Similarly, the impact of negative polarity on the visual resolution stayed significant without color element [Friedman's two-way analysis of variance test: χ ^2^ = 13.43, *P* < 0.05].

Linear regression was run to understand how the contrast ratio predicts the polarity and color element in visual resolution measurements. A mean and standard deviation of contrast ratio against visual resolution measurements (logMAR) with superimposed regression line was plotted with and without a color background in Figure 2. Visual inspection of these two plots indicated a linear relationship between the variables. The prediction equation was: 


$$\text{With background color: }y=-0.3423x+0.4351,R^{2}=0.7742$$

$$\text{Without background color:}𝑦=-0.1349𝑥+0.2691,{𝑅}^{2}=0.8073$$

Contrast ratio accounts for 77.4% of the variation in visual resolution measurement with color background [F ${}_{\left(1,6\right)}$ = 20.76, *P* < 0.01]. Contrast ratio accounts for 97.16% of the variation in visual resolution measurements without color background [F ${}_{\left(1,6\right)}$ = 205.63, *P* < 0.01].

##  DISCUSSION

This study investigated how contrast ratio affects the polarity and color elements in visual resolution measurements. Our findings show that adding color to the background enhances resolution thresholds that hold only for contrast levels of 0.3 and higher. In comparison to earlier findings, the enhancement in the resolution threshold was found at much higher contrast levels of 0.5 and higher.^[30]^ In the case of positive polarity (black text), the present experiment reveals that the visual resolution measurements (as indicated by the logMAR scores obtained) worsen as the contrast decreases in both conditions with and without color factors. The comparison between these two conditions (with and without color factors) shows that visual resolution reduces at the lower contrast level of 0.15 as compared to the higher contrast level of 0.3.

The visual resolution readings exhibit approximately two lines of reduction at 0.15 contrast level with background color (0.43 logMAR) in comparison to the background without color (0.29 logMAR).

The chart used is similar to the Smith-Kettlewell Institute Low Luminance (SKILL) card test: both are designed with similar chart progression and multiple levels of background luminance.^[31]^ However, the SKILL card test was intended for a viewing distance of 40 cm, whereas the current study tested for a distance of 4 m. Nevertheless, both studies seem to agree that in visually normal persons, darker chart (reduced luminance at 15% contrast for the current study and 14% contrast for SKILL's study) acuity is responsible for high variance in the logMAR score. In comparison, high-contrast acuity contributes to lesser variance (better logMAR). The average recommended background luminance for standard VA charts is 85 cd/m^2^ to 300 cd/m^2^.^[32,33]^ However, the background luminance was unable to be set at the recommended value in current study due to color element. Therefore, incorporating color elements in any road signs should be done with caution. The reduced light level used in the current study can be likened to what is experienced by motorists driving at twilight.

Presentation of dark letters (indicated by lower luminance) on a light background (marked by higher luminance) is usually referred to as positive polarity, as opposed to negative polarity with light letters or symbols on a dark background.^[19]^ Positive polarity is the preferred choice of the print construct, as evidenced by its abundant use in books, journals, newspapers, and printed office documents.

A significant reduction in resolution was observed when shorter color wavelengths were applied at much lower contrast (0.15) as the chart's background color. However, the reduction is only significant in the black text but not in the white text in our study.

Our findings further suggest that black text used against shorter wavelengths (blue) is harder to resolve by human eyes than is white text. These results are consistent with those of an earlier study that found when the color blue was applied as a background color, it reduced visual performance.^[21]^ This assessment further suggests that larger-scale mechanisms are needed for color-based information processing rather than for luminance-based processing. Our study only examined four colors. The color characteristics could be examined from different perspective like hues, correlated color temperature, color rendering index, and spectral power distribution variation.

Our data without color factor are consistent with a previous study that reported that the luminance factor that contributes to the contrast ratio on its own was sufficient for reading rate and shape detection.^[7,34]^ Visual resolution is controlled by the resultant luminance fluctuation between text and background, influencing the contrast ratio. Our combine findings with and without color factors further support the earlier study that discovered visual acuity for achromatic conditions was better than that for color conditions.^[35]^

This was evidently due to the lack of neural high-pass filtering in the color system as compared to the luminance system.^[36]^ Previous studies found that contrast sensitivity was about two times better for luminance-modulated gratings than chromatic gratings at all spatial frequencies. Current findings also accord with earlier studies in that we discovered that acuity with a grayscale background is more accurate as it yields better logMAR than a colored background.^[5]^ The impact of the color element was further investigated and comparisons were made between the two text polarities within our study. We found that black text displays more accurate visual resolution than the white text under luminance influence. A study on contrast polarity and myopia development revealed that the choroid became about 16 µm thinner in only 1 hr when subjects read black text on a white background.^[37]^ In comparison, the choroid became about 10 µm thicker when subjects read white text on a black background.^[37]^ They further suggested that reading white text on a black screen or tablet might be a way to inhibit myopia, while conventional black text on a white background might stimulate myopia.^[37]^

In summary, our study reveals that as contrast decreases, color plays a more significant role than the non-color factor in both polarities in the resolution of fine detail as it influences the visual resolution outcome, which is reflected by the results of the poorer logMAR. Although our study was successful in determining the impact of color and polarity on visual resolution, there were two limitations to our study. Our subjects were tested with their habitual visual acuity and natural pupil. We were unable to control the chromatic aberration because we test our subjects at their habitual visual acuity and natural pupils. Future research is recommended to study the impact of chromatic aberrations on color visual resolution. Our sample only evaluated young adults. Hence, application of these results in assessing alternate age demographics may be incongruous. Additional research is required to consider the impact of age-related variations on the visual resolution in color and polarity.

##  Financial Support and Sponsorship

This work was supported by [600-IRMI 5/3/GIP (079/2019)].

##  Conflicts of Interest

Authors declare that there are no conflicts of interest
